# High-dimensional mass cytometry identifies T cell and B cell signatures predicting reduced risk of *Plasmodium vivax* malaria

**DOI:** 10.1172/jci.insight.148086

**Published:** 2021-07-22

**Authors:** Lisa J. Ioannidis, Halina M. Pietrzak, Ann Ly, Retno A.S. Utami, Emily M. Eriksson, Stephanie I. Studniberg, Waruni Abeysekera, Connie S.N. Li-Wai-Suen, Dylan Sheerin, Julie Healer, Agatha M. Puspitasari, Dwi Apriyanti, Farah N. Coutrier, Jeanne R. Poespoprodjo, Enny Kenangalem, Benediktus Andries, Pak Prayoga, Novita Sariyanti, Gordon K. Smyth, Leily Trianty, Alan F. Cowman, Ric N. Price, Rintis Noviyanti, Diana S. Hansen

**Affiliations:** 1 Walter and Eliza Hall Institute of Medical Research, Parkville, Victoria, Australia.; 2 Department of Medical Biology, The University of Melbourne, Parkville, Victoria, Australia.; 3 Eijkman Institute for Molecular Biology, Jakarta, Indonesia.; 4 Papuan Health and Community Development Foundation, Papua, Indonesia.; 5 School of Mathematics and Statistics, The University of Melbourne, Parkville, Victoria, Australia.; 6 Global and Tropical Health Division, Menzies School of Health Research and Charles Darwin University, Darwin, Northern Territory, Australia.; 7 Centre for Tropical Medicine and Global Health, Nuffield Department of Medicine, University of Oxford, Oxford, United Kingdom.; 8 Mahidol-Oxford Tropical Medicine Research Unit, Mahidol University, Bangkok, Thailand.

**Keywords:** Immunology, Infectious disease, Adaptive immunity, Malaria

## Abstract

IFN-γ–driven responses to malaria have been shown to modulate the development and function of T follicular helper (TFH) cells and memory B cells (MBCs), with conflicting evidence of their involvement in the induction of antibody responses required to achieve clinical immunity and their association with disease outcomes. Using high-dimensional single-cell mass cytometry, we identified distinct populations of TH1-polarized CD4^+^ T cells and MBCs expressing the TH1-defining transcription factor T-bet, associated with either increased or reduced risk of *Plasmodium vivax* (*P. vivax*) malaria, demonstrating that inflammatory responses to malaria are not universally detrimental for infection. Furthermore, we found that, whereas class-switched but not IgM^+^ MBCs were associated with a reduced risk of symptomatic malaria, populations of TH1 cells with a stem central memory phenotype, TH17 cells, and T regulatory cells were associated with protection from asymptomatic infection, suggesting that activation of cell-mediated immunity might also be required to control persistent *P*. *vivax* infection with low parasite burden.

## Graphical abstract

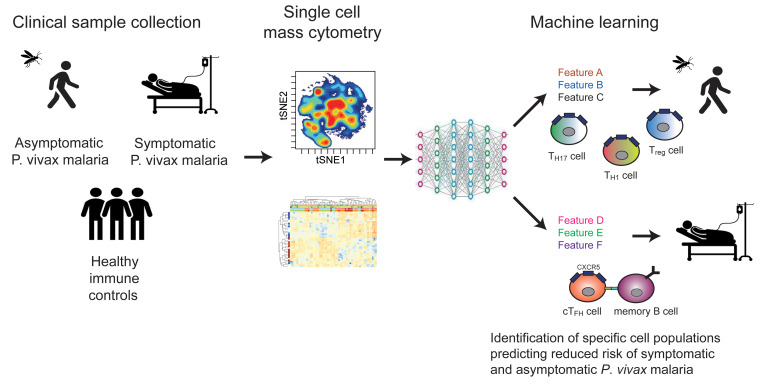

## Introduction

The World Health Organization estimates that 200 million clinical cases and 400,000 deaths were attributable to malaria in 2019 (1). However, due to extended periods of lockdown and overstretched government resources in response to the COVID-19 pandemic, many malaria-preventive measures were disrupted during 2020, raising concerns that the morbidity and mortality associated with malaria will climb to levels not seen for decades (2, 3). *Plasmodium falciparum* (*P. falciparum*) has been recognized as the most virulent species of malaria parasites. However, *Plasmodium vivax* (*P. vivax*) has a much wider geographical distribution, with 40% of the world’s population at risk of infection, and is responsible for a significant burden of disease in the Asia-Pacific, the Americas, and the Horn of Africa (4). The blood-stage form of the parasite is responsible for the symptoms of *P*. *vivax* infection, which range from a flu-like illness through to more severe complications, such as anemia, respiratory distress, acute kidney injury, multiorgan failure, and shock (5, 6). Young children with low levels of immunity are at greatest risk of symptomatic *P*. *vivax* malaria (7–9).

Naturally acquired immunity to *Plasmodium* parasites is often acquired after extended exposure to the parasite (10). This form of immunity is not sterilizing but prevents symptomatic episodes by substantially reducing parasite densities below the threshold capable of causing clinical illness (10). Compared with *P*. *falciparum*, much less is known about the acquisition of immunity to *P*. *vivax*. It has been suggested that immunity to *P*. *vivax* is acquired more rapidly compared with *P*. *falciparum* (9), and in endemic regions, morbidity peaks at an earlier age in *P*. *vivax*, with adults experiencing asymptomatic infection, consistent with the development of clinical immunity. In low-transmission settings, though, the risk of symptomatic *P*. *vivax* malaria does not appear to be dependent on age (11).

It is accepted that antibodies are a key component of clinical immunity to malaria. Seminal studies conducted decades ago established that passive transfer of immunoglobulin G (IgG) from clinically immune adults alleviates clinical symptoms and reduces parasite burden in nonimmune children (12, 13). Furthermore, antibodies against several blood-stage antigens have been found to be associated with protection from symptomatic *P*. *falciparum* and *P*. *vivax* malaria (14–19), with roles that include inhibition of parasite invasion into the red blood cell and opsonization of parasites for phagocytosis by effector cells (20, 21).

The acquisition of long-lived antibody-mediated immunity requires the establishment of germinal centers (GCs) in secondary lymphoid organs. In GCs, after interaction with cognate antigen, activated B cells undergo somatic hypermutation of their Ig genes followed by selection of B cell clones producing antibody with high affinity for antigen. GC function requires help from a subset of T cells named T follicular helper (TFH) cells (22). These cells orchestrate GC responses and promote the differentiation of naive B cells into long-lived plasma cells and memory B cells (MBCs), which circulate and become rapidly activated upon reencounter with their specific antigen (23).

Because of the pivotal role that TFH cells and MBCs play in the induction and recall of antibody responses, recent studies in mouse models and human infection investigated these populations to establish associations between their functional capacity and mechanisms underlying the slow acquisition of immunity to malaria. MBCs specific for several *P*. *vivax* and *P*. *falciparum* antigens have been observed in malaria-exposed individuals in different geographical regions (24–28). Although these cells were found to be short-lived in individuals residing in areas of high seasonal transmission (28), *P*. *vivax*–specific MBCs were stably maintained in individuals in areas of low malaria transmission that experience infrequent symptomatic malaria episodes (27). These observations suggest that the inflammatory responses contributing to clinical episodes of severe malaria have a detrimental effect on the development of humoral immunity. In support of this view, proinflammatory cytokines, such as IFN-γ, induced during symptomatic *P*. *berghei* infection in mice, were found to upregulate the expression of the T helper 1 (TH1) cell-defining transcription factor T-bet in TFH cells, which inhibits their differentiation and results in reduced GC responses to infection (29). Similarly, symptomatic *P*. *falciparum* and *P*. *vivax* malaria infections were found to induce the activation of circulating TH1-like TFH cells (30), with limited helper capacity (31), that were negatively associated with antibody responses to infection. While these studies support the idea that inflammatory responses induced by acute malaria have a detrimental impact on the T_FH_ pool, recent findings in mice identified TH1 memory CD4^+^ T cells induced in response to infection capable of successfully sustaining B cell help (32), thus challenging the notion that TH1-polarized CD4^+^ T cells impair the development of long-lived antibody-mediated immunity.

It is accepted that acute *P*. *falciparum* and *P*. *vivax* malaria infections induce high frequencies of CD27^–^CD21^–^ atypical MBCs (25, 33–37). Similar to circulating TFH cells observed in acute malaria, atypical MBCs also express variable levels of the TH1 transcription factor T-bet (34, 35, 38). The role of atypical MBCs in malaria is controversial. Whereas some studies propose that these cells are an important source of parasite-specific antibodies (39) and contribute to protection from infection (40–42), other studies have disputed the effector capacity of these cells (34, 36) and instead proposed associations between T-bet^+^ atypical MBCs and symptomatic malaria (38).

Most of our knowledge on immune responses to malaria originated from bulk population data in which cells from the same subtype are considered as a single unit and all members of the class are by definition homogeneous. These analyses often lead to the conflicting view that the same MBC and T cell responses associate with opposite infection outcomes (25, 38, 40, 41). Furthermore, despite the increasingly recognized burden that *P*. *vivax* poses to malaria elimination programs, due to the latent hypnozoite stage that results in relapsing infections in the absence of mosquito bites (43), the immunological processes underlying the acquisition of immunity to this parasite have only received marginal attention when compared with *P*. *falciparum* (25, 30, 37). To address these issues, we used high-dimensional single-cell mass cytometry to untangle the complexity of the MBC and CD4^+^ T cell response induced in response to *P*. *vivax* malaria. Our approach was able to dissect distinct TH1-polarized CD4^+^ T cells and T-bet^+^ subpopulations of atypical MBCs associated with either increased or reduced risk of *P*. *vivax* infections, supporting the notion that inflammatory responses to malaria are not unanimously associated with poor infection outcomes. Furthermore, while class-switched MBCs were identified as predictors of reduced risk of symptomatic malaria, specific subsets of CD4^+^ T cells with different polarization status appeared to be associated with protection from asymptomatic infection, suggesting that the activation of cell-mediated responses might be required for the control of these persistent infections of low parasite burden.

## Results

### Cohort characteristics.

**Figure 1:**
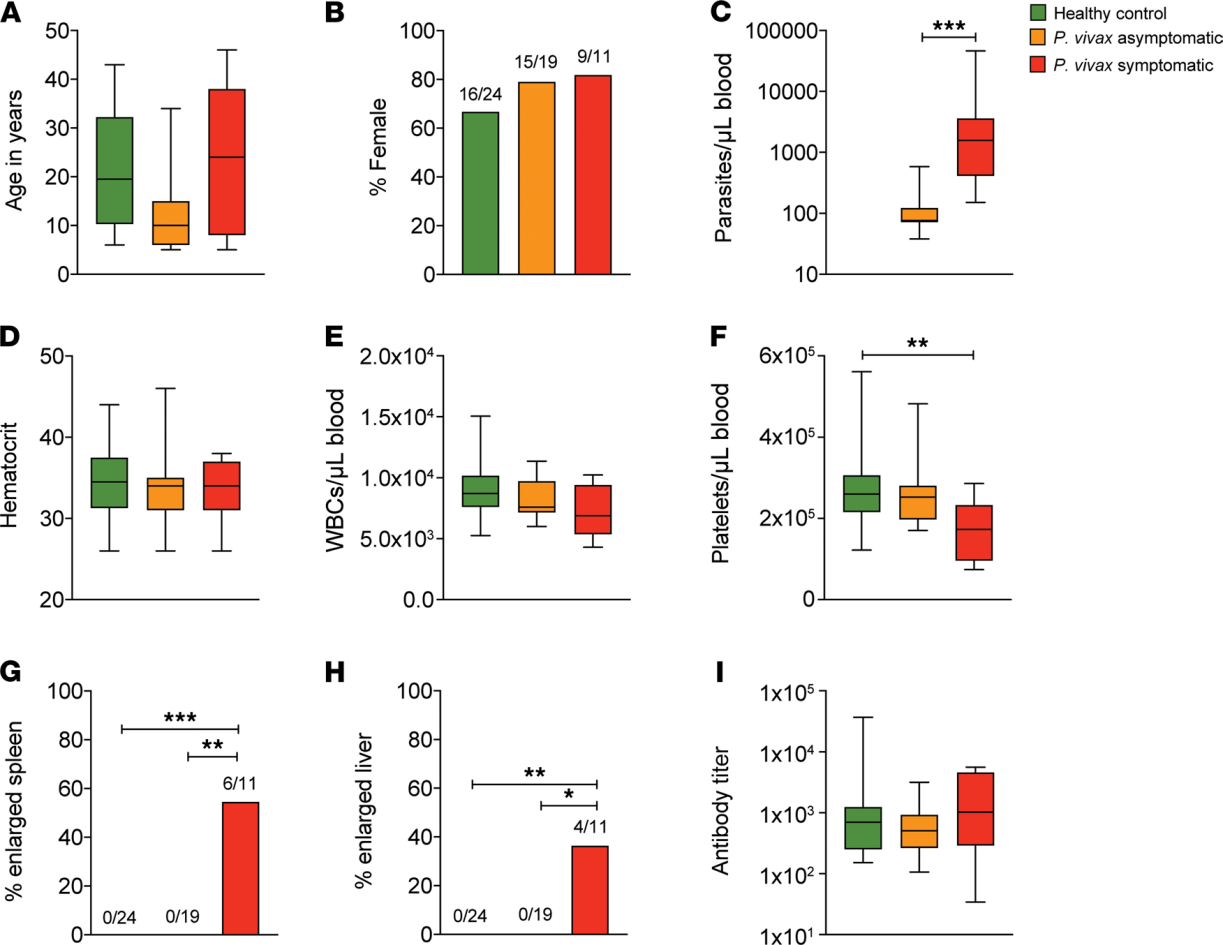
Study cohort characteristics. *P*. *vivax* symptomatic (*n* = 11) and asymptomatic (*n* = 19) infected individuals as well as healthy immune controls (*n* = 24) were selected for analysis of immune responses to infection. Age (**A**) and sex (**B**) were not different while parasitemia was significantly higher in the symptomatic group (**C**). Other clinical parameters determined in the study include hematocrit (**D**), WBC count (**E**), platelet count (**F**), as well as the proportion of participants presenting with enlarged spleen (**G**) or liver (**H**). Antibody titers specific for *P*. *vivax* recombinant DBP were determined by ELISA (**I**). Boxes represent the 25th to 75th percentile, whiskers show the range (minimum to maximum), and lines represent the median. *P < 0.05, **P < 0.01, ***P < 0.001. Significance was determined by Kruskal-Wallis with Dunn’s multiple-comparison test (**A**, **D**, **E**, **F**, and **I**), Mann-Whitney test (**C**), or Fisher’s exact test (**B**, **G**, and **H**).

The clinical characteristics of the cohort used in the study are summarized in Figure 1. In the Timika region of Indonesia, Papuans reside both in the lowlands, where malaria exposure is common, and the highlands, where malaria is absent. Migration of nonimmune adults from the highlands to lowlands means symptomatic infection can occur in all age groups. There was no difference in age or sex composition among the symptomatic, asymptomatic, and healthy control groups (Figure 1, A and B). As expected, the mean parasite density of individuals with a symptomatic infection was significantly higher than those with an asymptomatic infection (Figure 1C). No significant differences were found in hematocrit and white blood cell (WBC) counts at enrollment, while platelets were significantly lower in the symptomatic group compared with the healthy control group (Figure 1, D–F). Among individuals with a symptomatic infection, 54.5% presented with an enlarged spleen and 36.4% with an enlarged liver (Figure 1, G and H).

Duffy binding protein (DBP) is a key *P*. *vivax* invasion ligand and is a major antigenic target of naturally acquired immunity (44). Assessment of antibody responses to DBP confirmed that not only *P*. *vivax*–infected participants but also healthy controls had been previously exposed to *P*. *vivax*, with high DBP-specific IgG titers detected in all individuals from each group (Figure 1I). Thus, all individuals had been previously exposed to *P*. *vivax*, and healthy controls in the community possessed preexisting immunity to malaria.

### High-dimensional mass cytometry reveals multiple subsets of memory CD4^+^ T cells and MBCs after exposure to P. vivax.

To identify subpopulations of CD4^+^ T cells and MBCs associated with reduced risk of *P*. *vivax* malaria, PBMCs from symptomatic and asymptomatic *P*. *vivax*–infected individuals as well as healthy immune controls were stained with a panel of metal-labeled antibodies specific for a range of B cell and T cell markers (Supplemental Table 1; supplemental material available online with this article; https://doi.org/10.1172/jci.insight.148086DS1) and analyzed by mass cytometry. In an initial analysis, t-distributed stochastic neighbor embedding (t-SNE) was used to visualize the expression of markers across the entire MBC and CD4^+^ T cell compartments. Supplemental Figure 1A shows striking differences in the MBC composition of *P*. *vivax*–infected individuals and healthy immune controls. Whereas MBCs expressing CD19, CD20, CD21, CD27, CD45RA, IgD, IgM, CXCR5, CCR6, and T-bet were clearly identified, metal-labeled antibodies against IgG and Fc receptor-like 5 (FcRL5) showed poor reactivity and were able to detect only low numbers of cells across all groups (Supplemental Figure 1B). The same level of heterogeneity between infected individuals and healthy controls was observed across the memory CD4^+^ T cell pool, with good expression of CD3, CD4, CCR6, CXCR3, CXCR5, programmed cell death protein 1 (PD-1), T-bet, CD27, CD25, and CD127 across this compartment but only low recognition of ICOS (Supplemental Figure 1, C and D).

**Figure 2:**
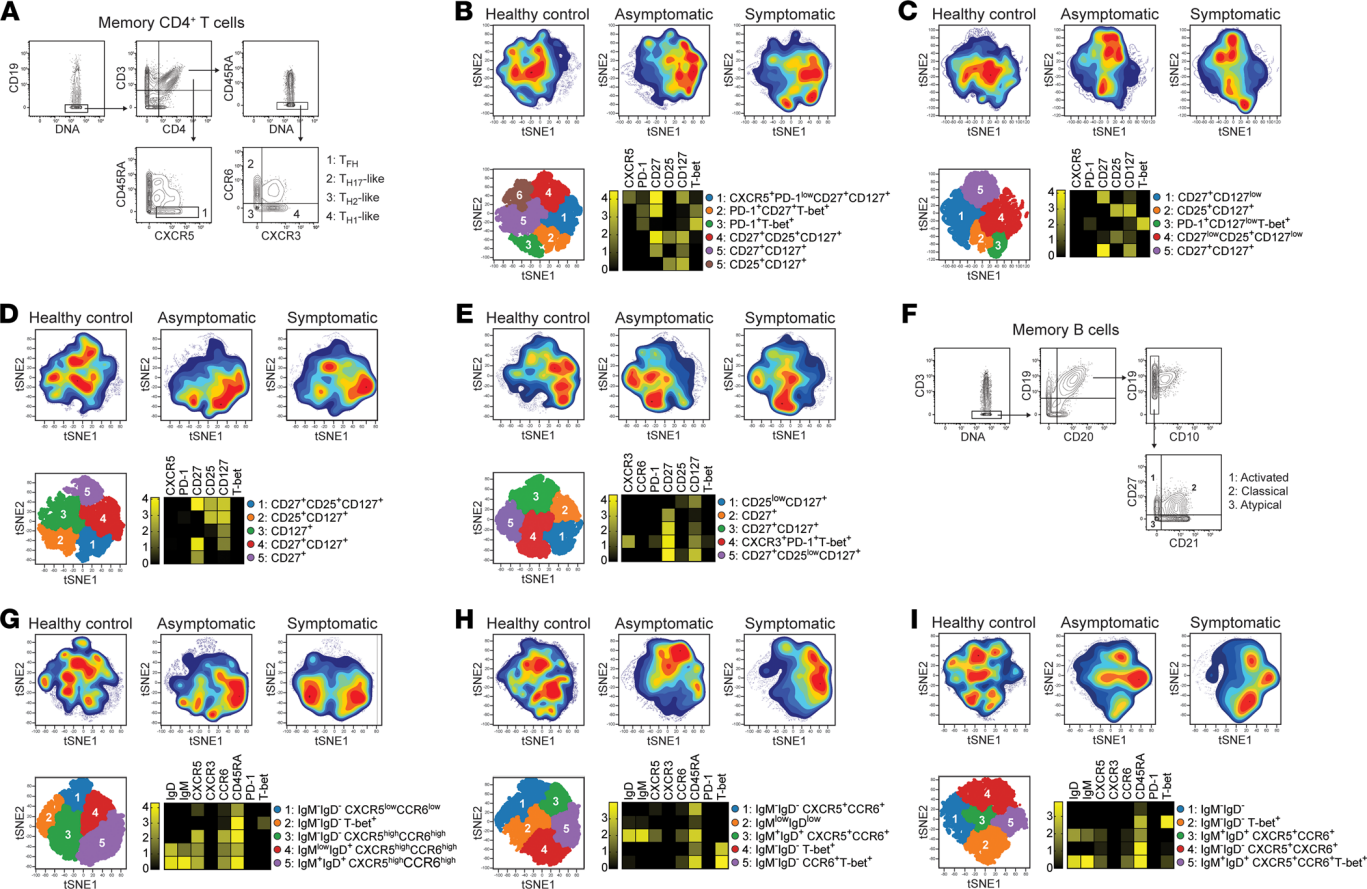
Identification of memory CD4^+^ T cell and MBC subpopulations induced after exposure to *P. vivax.* PBMCs from *P*. *vivax* symptomatic (*n* = 11) and asymptomatic (*n* = 19) infected individuals as well as healthy immune controls (*n* = 24) were stained with a panel of metal-labeled antibodies and analyzed by cytometry by time of flight (CyTOF). Manual gating was used to select individual memory CD4^+^ T cell (**A**) and MBC populations (**F**). t-SNE analysis was then performed on TH1-like memory CD4^+^ T cells (CD19^–^CD3^+^CD4+CD45RA^–^CCR6^–^CXCR3^+^; **B**), TH2-like memory CD4^+^ T cells (CD19^–^CD3^+^CD4^+^CD45RA^–^CCR6^–^CXCR3^–^; **C**), TH17-like memory CD4^+^ T cells (CD19^–^CD3^+^CD4^+^CD45RA^–^CCR6^+^CXCR3^–^; **D**), circulating memory TFH cells (CD19^–^CD3^+^CD4^+^CD45RA^–^CXCR5^+^; **E**), classical MBCs (CD3^–^CD19^+^CD20^+^CD10^–^CD27^+^CD21^+^; **G**), atypical MBCs (CD3^–^CD19^+^CD20^+^CD10^–^CD27^–^CD21^–^; **H**) and activated MBCs (CD3^–^CD19^+^CD20^+^CD10^–^CD27^+^CD21^–^; **I**), and FlowSOM clustering was used to identify individual cell populations. The t-SNE plots in the top panel display cell density and represent the pooled data for each group, while the lower panel shows a projection of the FlowSOM clusters on a t-SNE plot. Heatmaps show the median marker expression for each FlowSOM cluster.

Within the circulating memory CD4^+^ T cell pool, the expression of the chemokine receptors CXCR3 and CCR6 allowed us to distinguish TH1-like CD4^+^ T cells (CXCR3^+^CCR6^–^), TH2-like CD4^+^ T cells (CXCR3^–^CCR6^–^), and TH17-like CD4^+^ T cells (CXCR3^–^CR6^+^; Figure 2A). To explore the composition of these populations as well as CXCR5^+^ circulating memory TFH cells, FlowSOM clustering was performed, and marker expression was assessed within each subpopulation. This approach revealed a high level of heterogeneity within each cell population and allowed the identification of 5–6 subpopulations of TH1-like memory CD4^+^ T cells, TH2 memory CD4^+^ T cells, TH17 memory CD4^+^ T cells, and memory TFH cells (Figure 2, B–E). Within each compartment, various clusters of T cells expressing variable levels of CD27, CD127, and CD25 were clearly identified (Figure 2, B–E). Interestingly, clusters of T-bet^+^ cells were found among some but not all CXCR3^+^ TH1-like CD4^+^ T cells (Figure 2B). Clusters of PD-1^+^ cells could be found among TH1-like and TH2-like CD4^+^ T cells, although the expression level of this marker was in general low (Figure 2B). One subset of T-bet^+^CXCR3^+^ and various clusters of CXCR3^–^CCR6^–^ cells were detected among the circulating TFH pool (Figure 2E).

CD21 and CD27 expression allows the identification of CD21^+^CD27^+^ classical MBCs, CD21^–^CD27^–^ atypical MBCs, and CD21^–^CD27^+^ activated MBCs (Figure 2F). FlowSOM clustering was applied to reveal the complexity within each of these MBC pools (Figure 2, G–I). Due to the low reactivity of metal-labeled antibodies against IgG and FcRL5 (Supplemental Figure 1), these markers were not included in this analysis. The majority of classical MBC clusters that were identified expressed high levels of CD45RA and variable levels of the chemokine receptors CXCR5 and CCR6 but differed in their expression of surface immunoglobulins (Figure 2G). In contrast, most atypical and activated MBC subpopulations expressed few if any of these chemokine receptors (Figure 2, H and I). Both IgM^+^IgD^+^ as well as class-switched cells were found in these pools, with 2 clusters of IgM^–^IgD^–^ atypical MBCs and 1 population of IgM^–^IgD^–^ activated MBCs expressing high levels of T-bet.

**Figure 3:**
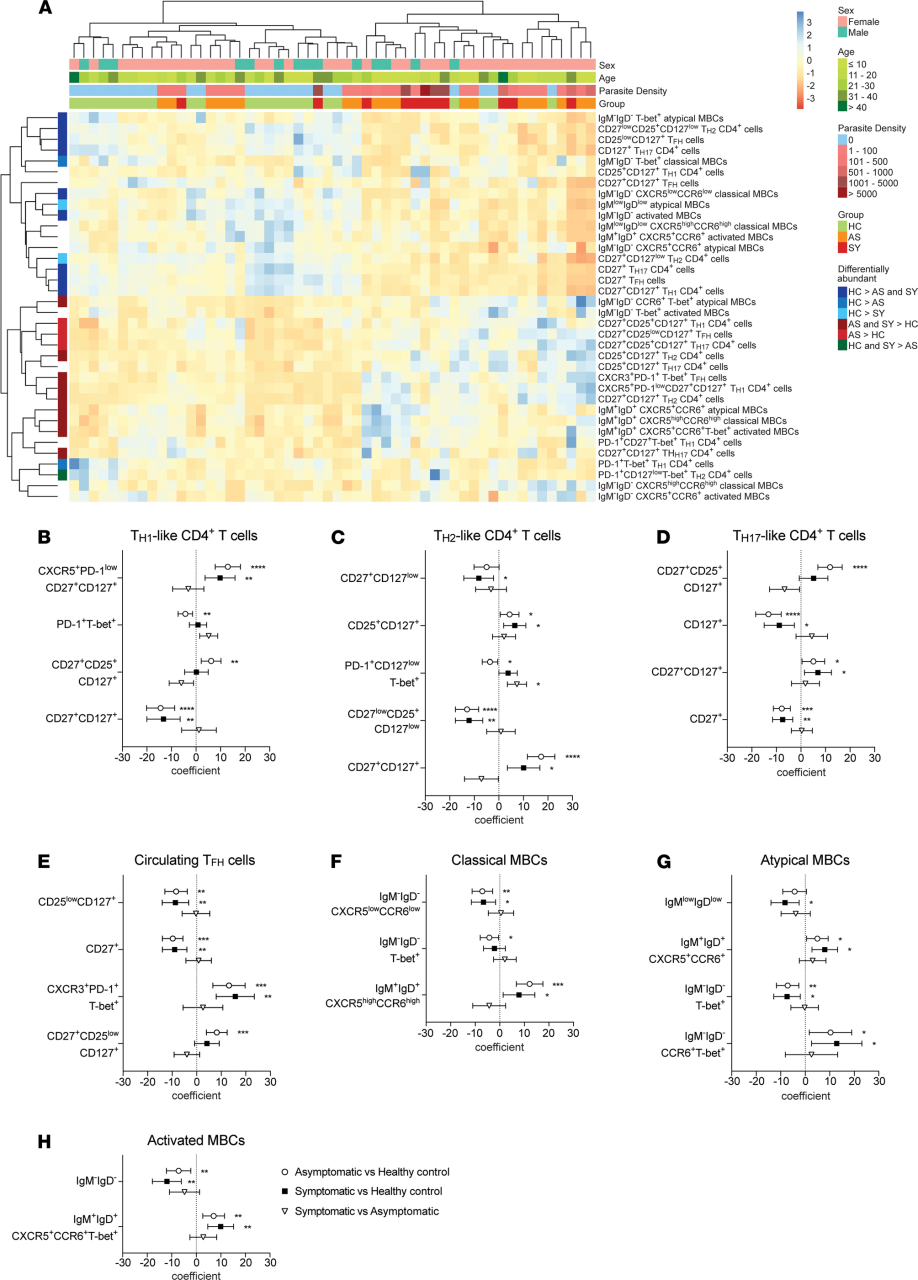
Identification of differentially abundant memory CD4^+^ T cell and MBC subpopulations after exposure to *P. vivax.* PBMCs from *P*. *vivax* symptomatic (*n* = 11) and asymptomatic (*n* = 19) infected individuals as well as healthy immune controls (*n* = 24) were stained with a panel of metal-labeled antibodies and analyzed by CyTOF. The frequency of each cell population identified by FlowSOM analysis was determined, and linear regression models were fitted to identify differentially abundant cell populations. Unsupervised hierarchical clustering heatmap showing the frequency of each cell population for each participant (**A**). Differentially abundant TH1-like CD4^+^ T cell (**B**), TH2-like CD4^+^ T cell (**C**), TH17-like CD4^+^ T cell (**D**), circulating TFH cell (**E**), classical MBC (**F**), atypical MBC (**G**), and activated MBC (**H**) subpopulations identified by linear regression. Regression coefficients represent log_2_ fold changes between groups. Symbols represent the estimated value and vertical lines depict the 95% confidence interval. *False discovery rate (FDR) < 0.05, **FDR < 0.01, ***FDR < 0.001, ****FDR < 0.0001. HC, healthy control; AS, asymptomatic; SY, symptomatic.

The percentage of each B cell and T cell cluster was determined, and linear regression models were applied to identify differentially abundant subpopulations among groups. Initial unsupervised hierarchical clustering of study participants based on all cellular populations identified by FlowSOM analysis segregated most immune healthy community controls from *P*. *vivax*–infected symptomatic and asymptomatic individuals as shown in Figure 3A. Linear regression models identified 26 differentially abundant subpopulations among groups, with the vast majority of the differences observed between healthy immune controls and both symptomatic and asymptomatic *P*. *vivax*–infected participants. In agreement with previous reports (29, 31), a cluster of CXCR5^+^PD-1^lo^CD27^+^CD127^+^ TH1-like CD4^+^ T cells, consistent with a TFH cell phenotype, was significantly more abundant in both symptomatic and asymptomatic *P*. *vivax*–infected individuals compared with healthy community controls (Figure 3B). In contrast, a subset of CD27^+^CD127^+^ TH1-like memory CD4^+^ T cells was significantly reduced in infected individuals, relative to healthy controls (Figure 2B). A similar cluster of CD27^+^CD127^+^ memory CD4^+^ T cells was also differentially abundant within the TH2-like and TH17-like pools (Figure 3, C and D), but in those cases, these cells were significantly higher in study participants carrying symptomatic and asymptomatic *P*. *vivax* infections. Conversely, a cluster of TH2-like CD27^lo^CD25^+^CD127^lo^ and 2 clusters of TH17-like CD27^+^ and TH17-like CD127^+^ cells were underrepresented in infected individuals relative to immune healthy controls (Figure 3, C and D). Together, these results suggest that the CD4^+^ T cell pool induced in response to *P*. *vivax* infection is heterogeneous and that subpopulations that differ in their helper polarization status associate with the same infection outcome. Confirming our findings in Figure 3B, increased abundance of TH1-like CXCR3^+^PD-1^+^T-bet^+^ cells was found among infected individuals, when gated circulating memory TFH cells were analyzed (Figure 3E). In contrast, clusters of CXCR3^–^CCR6^–^ TH2-like CD25^lo^CD127^+^ and CD27^+^ TFH cells were significantly reduced upon infection compared with healthy immune controls (Figure 3E).

Linear regression analysis of the MBC compartment revealed that whereas class-switched classical MBCs expressing low levels of CXCR5 and CCR6 were overrepresented in healthy immune controls relative to *P*. *vivax* symptomatic and asymptomatic infected participants, their IgM^+^IgD^+^ counterparts, expressing high levels of these chemokine receptors, were more abundant in individuals carrying an infection (Figure 3F). Clusters displaying a similar phenotype were also significantly more abundant among infected individuals in the atypical and activated MBC compartments (Figure 3, G and H). Thus, an abundance of unswitched IgM^+^IgD^+^ MBCs appears to be a feature of concomitant *P*. *vivax* infection. Interestingly, 2 populations of T-bet^+^, class-switched, atypical MBCs were identified by our analysis. While CCR6^+^ cells were significantly more abundant in response to infection, CCR6^–^ atypical MBCs were significantly higher in healthy immune controls relative to infected individuals (Figure 3G). Thus, these results revealed the presence of diverse atypical MBCs subsets in the blood of *P*. *vivax*–infected individuals that appear to be associated with different infection outcomes.

### Diverse CD4^+^ T cell populations with different polarization status circulate in response to P. vivax malaria.

**Figure 4:**
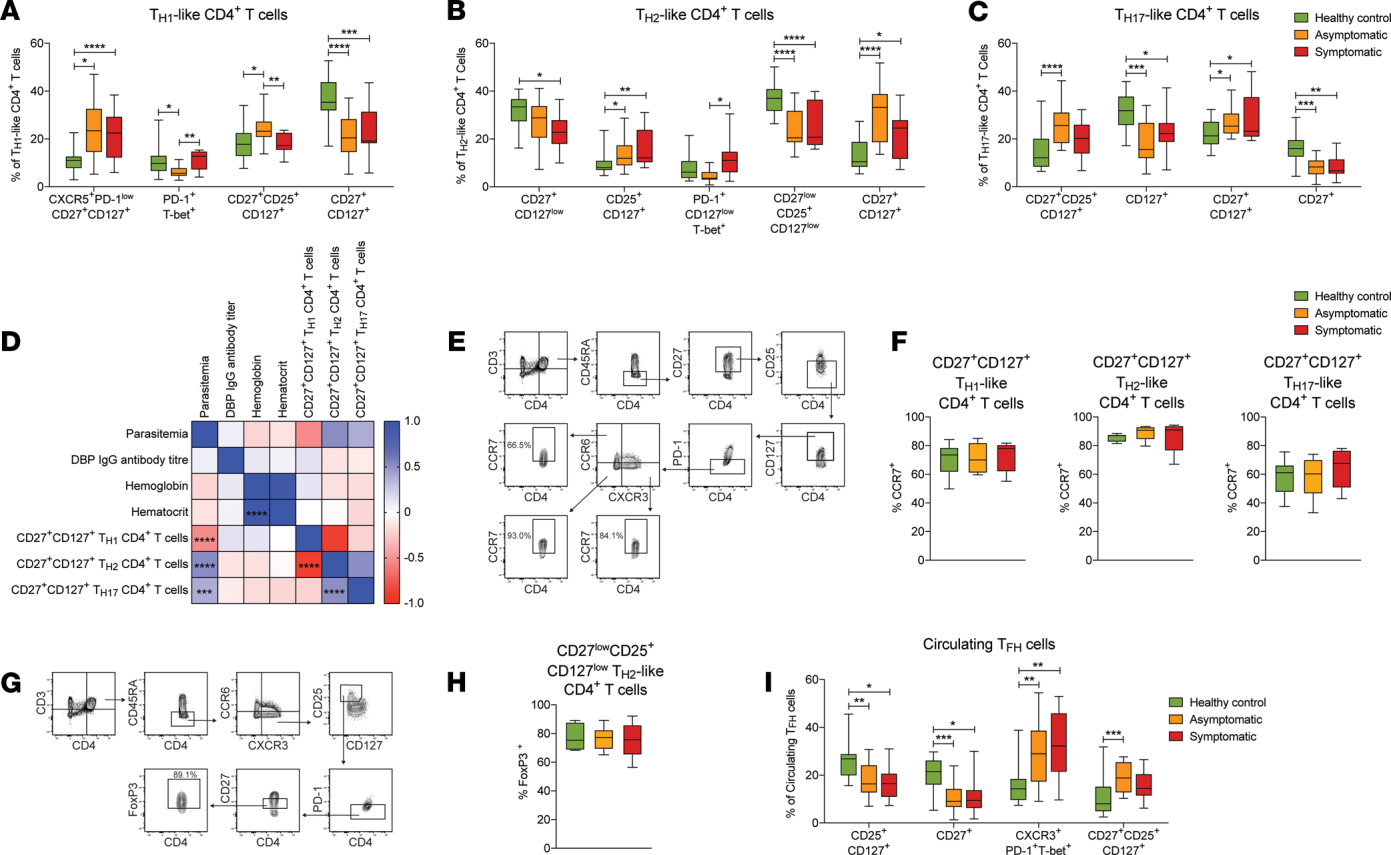
CD4^+^ T cell populations with different polarization circulate in response to *P. vivax* malaria. PBMCs from *P*. *vivax* symptomatic (*n* = 11) and asymptomatic (*n* = 19) infected individuals as well as healthy immune controls (*n* = 24) were stained with a panel of metal-labeled antibodies and analyzed by CyTOF. Percentages of TH1-like CD4^+^ T cell (**A**), TH2-like CD4^+^ T cell (**B**), TH17-like CD4^+^ T cell (**C**), and circulating TFH cell subpopulations (**I**) identified by CyTOF after FlowSOM analysis. Boxes represent the 25th to 75th percentile, whiskers show the range (minimum to maximum) and lines represent the median. The relationship between CD27^+^CD127^+^ TH1-, TH2-, and TH17-like CD4^+^ T cells and clinical parameters was determined by Spearman’s rank correlation (**D**). PBMCs from *P*. *vivax* symptomatic (*n* = 6) and asymptomatic (*n* = 8) infected individuals as well as healthy immune controls (*n* = 6) were stained with fluorescent antibodies and analyzed by flow cytometry to assess CCR7 expression among CD27^+^CD127^+^ TH1-, TH2-, and TH17-like CD4^+^ T cells (**E** and **F**) and FoxP3 expression among CD27^lo^CD25^+^CD127^lo^ TH2-like CD4^+^ T cells (**G** and **H**). Representative contour plots are shown. Boxes represent the 25th–75th percentile, whiskers show the range (minimum to maximum), and lines represent the median. *P < 0.05, **P < 0.01, ***P < 0.001, ****P < 0.0001. Significance was determined by 1-way ANOVA with Holm-Sidak posttest or Kruskal-Wallis with Dunn’s multiple-comparison test (**A**–**C**, **F**, **H**, and **I**) or Spearman’s rank correlation (**D**).

The relative frequency of CD4^+^ T cell subsets differentially abundant between *P*. *vivax*–infected individuals and healthy immune controls is shown in Figure 4. CD27^+^CD127^+^CD4^+^ T cells were abundant and identified across all TH pools (Figure 4, A–C), with increased percentages of TH1-polarized cells and reduced percentages of TH2- and TH17-polarized cells in healthy immune controls compared with *P*. *vivax* symptomatic and asymptomatic infected individuals. Furthermore, frequencies of TH1- and TH2-polarized cells were negatively and positively correlated with parasitemia, respectively, and negatively associated with each other (Figure 4D). CD27^+^CD127^+^CD4^+^ T cells have been previously found in other infection settings (45, 46) and appear to have features of stem-like central memory T (TSCM) cells with self-renewal capacity. To examine if this was the case for *P*. *vivax*–exposed individuals, the expression of the central memory homing receptor CCR7 was examined by flow cytometry among gated CD27^+^CD127^+^CD25^–^PD-1^–^CD4^+^ T cells (Figure 4, E and F). We found that while approximately 80% of TH1- and TH2-like cells expressed CCR7, around 60% of TH17-polarized cells expressed this receptor and were therefore consistent with a CD4^+^ TSCM-like phenotype (Figure 4F).

Within the TH2-like memory CD4^+^ T cell pool (Figure 4B), a population of CD27^lo^CD127^lo^CD25^+^ cells, resembling regulatory T cells (Tregs), was significantly higher in healthy immune controls compared with infected individuals. To confirm this, the expression of the Treg-defining transcription factor FoxP3 was examined in these cells by flow cytometry (Figure 4, G and H). We found that over 80% of CD27^lo^CD127^lo^CD25^+^ cells readily expressed FoxP3 (Figure 4H).

CXCR3^+^ TH1-like TFH cells were found to be highly abundant not only in symptomatic but also in asymptomatic infected individuals (Figure 4I). In contrast, populations of TH2-like TFH cells expressing high levels of CD27 and CD127 were significantly increased in healthy immune controls compared with *P*. *vivax*–infected individuals. Thus, together these results indicate that high frequencies of CXCR3^+^ TFH cells along with reduced TH2-like TFH cell, TH1 TSCM, and Treg compartments are the main features of the circulating memory CD4^+^ T cell pool in *P*. *vivax*–infected individuals.

### Subsets of classical and atypical class-switched MBCs associate with reduced P. vivax parasite burden.

**Figure 5:**
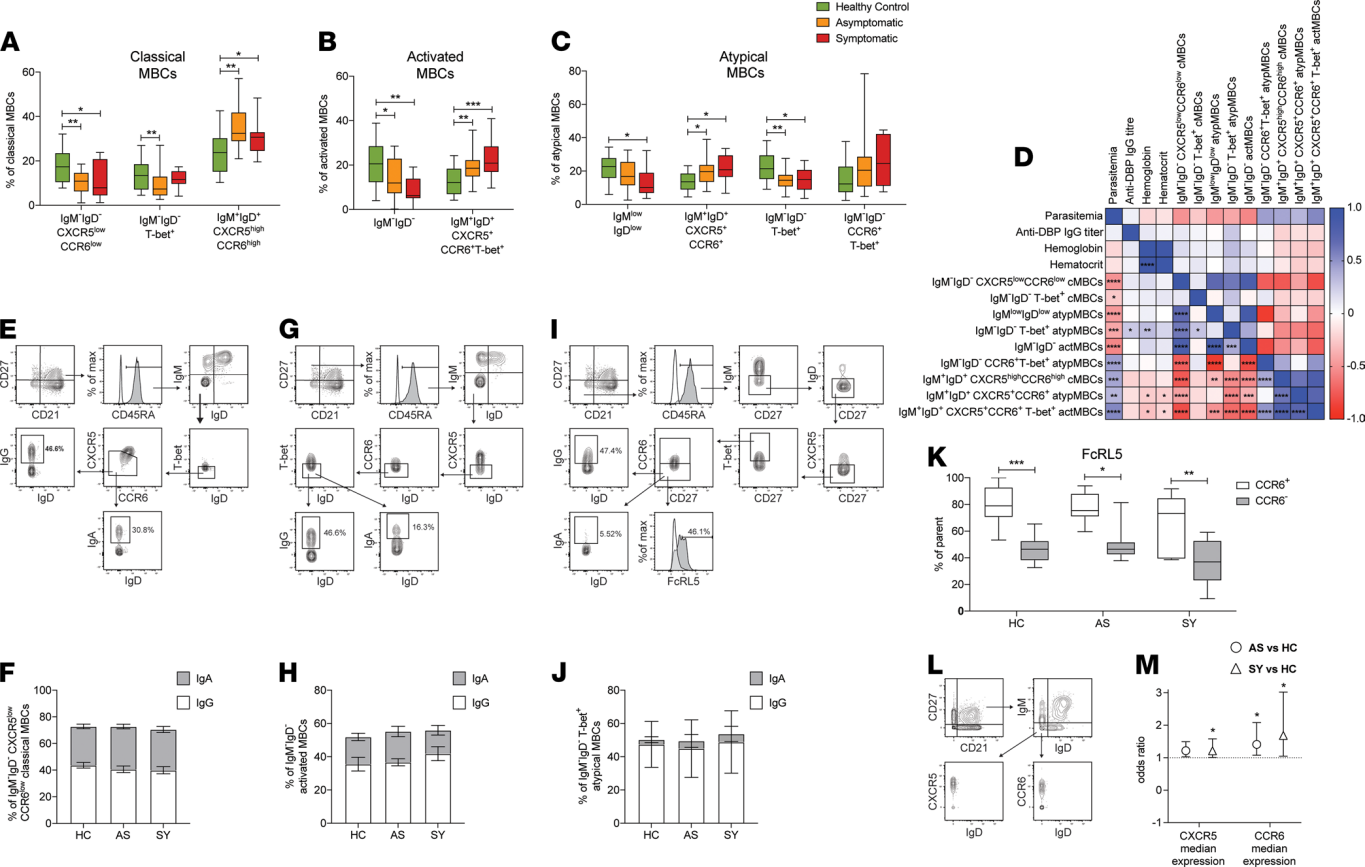
Subsets of classical and atypical class-switched MBCs associate with reduced *P. vivax* parasite burden. PBMCs from *P*. *vivax* symptomatic (*n* = 11) and asymptomatic (*n* = 19) infected individuals as well as healthy immune controls (*n* = 24) were stained with a panel of metal-labeled antibodies and analyzed by CyTOF. Percentages of classical MBC (**A**), activated MBC (**B**), and atypical MBC (**C**) subpopulations identified by CyTOF after FlowSOM analysis. Boxes represent the 25th to 75th percentile, whiskers show the range (minimum to maximum), and lines represent the median. The relationship between MBC subpopulations and clinical parameters was determined by Spearman’s rank correlation (**D**). *P*. *vivax* symptomatic (*n* = 6) and asymptomatic (*n* = 8) infected individuals as well as healthy immune controls (*n* = 6) were stained with fluorescent antibodies and analyzed by flow cytometry to assess IgG and IgA expression among IgM^–^IgD^–^CXCR5^lo^CCR6^lo^ classical MBCs (**E** and **F**), IgM^–^IgD^–^ activated MBCs (**G** and **H**) and IgM^–^IgD^–^T-bet^+^ atypical MBCs (**I** and **J**) as well as FcRL5 expression among IgM^–^IgD^–^CCR6^+^T-bet^+^ atypical MBCs (**K**). Representative histograms and contour plots are shown. Stacked bars represent the mean ± SEM. Boxes represent the 25th to 75th percentile, whiskers show the range (minimum to maximum), and lines represent the median. CXCR5 and CCR6 expression was also assessed among gated IgM^–^IgD^–^ classical MBCs (**L**), and a logistic regression model was used to determine the association between CXCR5 and CCR6 expression levels and the risk of asymptomatic or symptomatic *P*. *vivax* infection (**M**). Symbols represent the odds ratio and vertical lines depict the 95% confidence interval; *P < 0.05, **P < 0.01, ***P < 0.001. Significance was determined by 1-way ANOVA with Holm-Sidak post test or Kruskal-Wallis with Dunn’s multiple-comparison post test (**A**–**C**, **F**, and **H**), Spearman’s rank correlation (**D**), or unpaired, 2-tailed *t* test or Mann-Whitney *U* test (**K**). HC, healthy control; AS, asymptomatic; SY, symptomatic.

Figure 5 depicts the relative frequencies of MBC subsets differentially abundant between immune healthy controls and *P. vivax*–infected individuals. Frequencies of IgM^+^IgD^+^ MBCs expressing high levels of the chemokine receptors CXCR5 and CCR6 were significantly higher in both *P*. *vivax* symptomatic and asymptomatic individuals compared with healthy controls within the classical and atypical compartments (Figure 5, A and B). A similar population of these cells that showed positive expression of T-bet was also found to be significantly higher in *P*. *vivax*–infected individuals within the activated MBC pool (Figure 5B). Interestingly, all these populations were tightly correlated with each other and showed positive correlations with parasitemia, while being negatively associated with hematocrit and hemoglobin levels (Figure 5D).

In general, IgM^+^IgD^+^ cells were negatively correlated with class-switched populations of MBCs (Figure 5D). Among those, a population of classical MBCs expressing low levels CXCR5 and CCR6 was significantly higher among healthy immune controls compared with both symptomatic and asymptomatic *P*. *vivax*–infected individuals (Figure 5A). Flow cytometry analysis revealed that these cells express IgG and IgA (Figure 5F) but not IgE (data not shown). The frequency of these cells correlated with other populations of class-switched cells within the activated and atypical MBC pools (Figure 5D). The majority of class-switched activated (Figure 5, G and H) and CCR6^–^T-bet^+^ atypical MBCs (Figure 5, I and J) expressed IgG and only low frequencies of IgA (Figure 5, G–J). Most of the class-switched MBC populations were negatively correlated with parasite densities (Figure 5D). Furthermore, high frequencies of CCR6^–^T-bet^+^ class-switched atypical MBCs were also associated with high parasite-specific IgG responses to infection and higher hemoglobin levels, suggesting an involvement for these cells in favorable disease outcomes (Figure 5D). In marked contrast CCR6^+^T-bet^+^, class-switched, atypical MBCs were significantly upregulated in both *P*. *vivax* symptomatic and asymptomatic infected individuals and showed strong correlations with parasite burden (Figure 5D). Furthermore, flow cytometry analysis revealed that the expression levels of the FcRL5 inhibitory receptor were significantly higher among CCR6^+^T-bet^+^, class-switched, atypical MBCs, compared with their CCR6^–^ counterparts (Figure 5K). Thus, together these results indicate that class switching and low expression of the chemokine receptors CXCR5 and CCR6 in MBCs rather than T-bet expression are associated with improved *P*. *vivax* infection outcomes. To further investigate this proposition, CXCR5 and CCR6 median expression in classical MBCs was determined (Figure 5L) for the study participants, and logistic regression models were applied to estimate odds ratios (Figure 5M). This analysis revealed that increased expression of CXCR5 and CCR6 on MBCs predicts increased risk of symptomatic *P*. *vivax* malaria.

### CD4^+^ T cell populations from diverse lineages and class-switched MBCs predict reduced risk of P.

**Figure 6:**
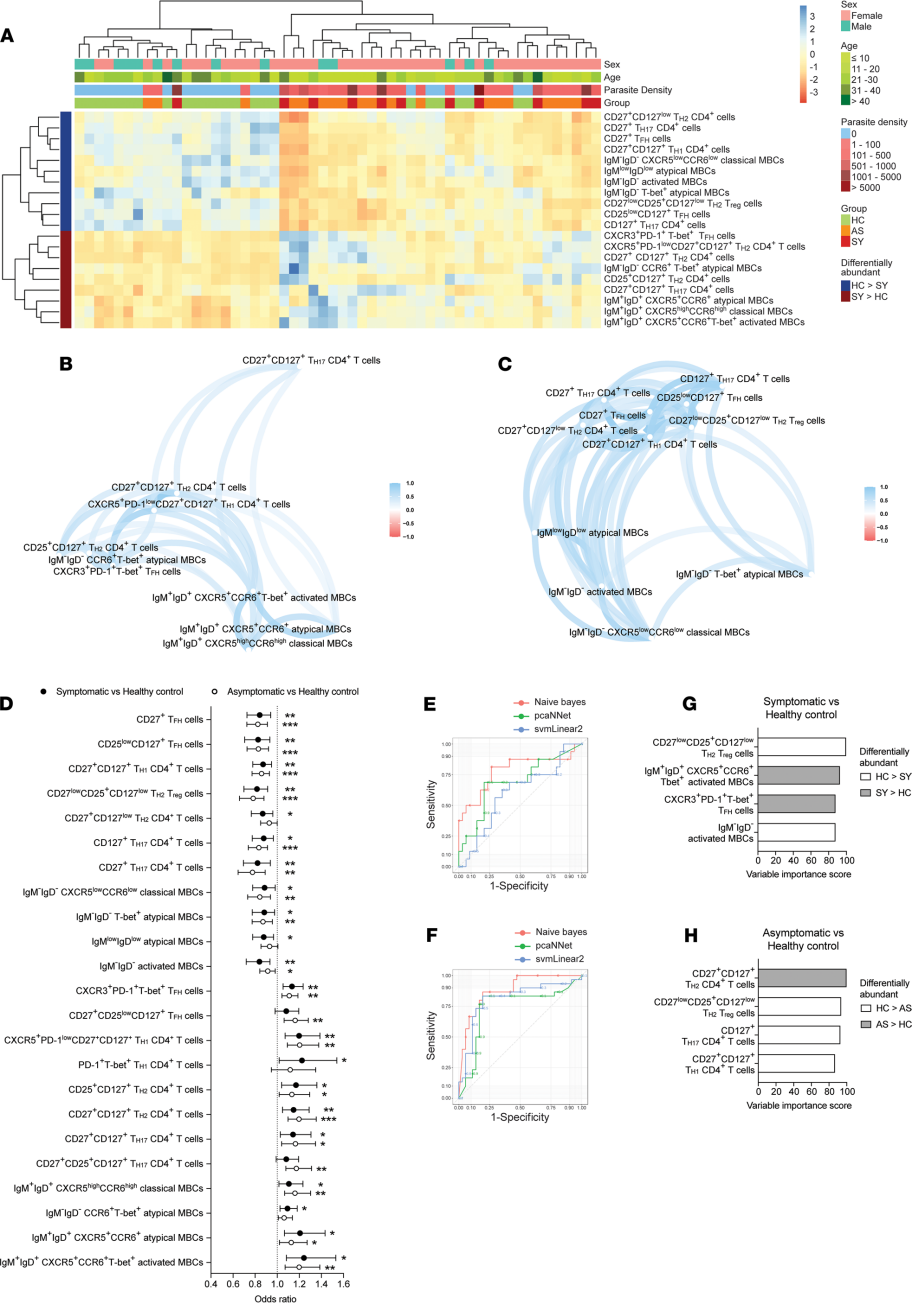
Memory CD4^+^ T cell population from diverse lineages and class-switched MBCs predict reduced risk of *P. vivax* malaria. PBMCs from *P*. *vivax* symptomatic (*n* = 11) and asymptomatic (*n* = 19) infected individuals as well as healthy immune controls (*n* = 24) were stained with a panel of metal-labeled antibodies and analyzed by CyTOF. Unsupervised hierarchical clustering heatmap showing the frequency of cell populations that were differentially abundant between healthy immune controls and symptomatic *P*. *vivax*–infected individuals (**A**). Spearman’s correlation networks were used to examine the relationship between cell populations that were either reduced (**B**) or increased (**C**) in healthy immune controls compared with individuals with a symptomatic infection. Logistic regression models were used to determine the association between cell frequencies and the risk of asymptomatic or symptomatic *P*. *vivax* infection (**D**). Symbols represent the odds ratio and vertical lines depict the 95% confidence interval. *P < 0.05, **P < 0.01, ***P < 0.001, ****P *<* 0.0001. The automated machine learning workflow SIMON was used to identify cell populations that best predict protection from both asymptomatic and symptomatic infection. Receiver operating characteristic (ROC) curves for classifying individuals with either a symptomatic (**E**) or asymptomatic (**F**) infection based on the relative frequency of each cell population. Colored curves represent the 3 best performing models. Top-ranked features (variable importance score > 85%) of symptomatic (**G**) and asymptomatic infection (**H**). Bars represent the variable importance score for each feature. HC, healthy control; AS, asymptomatic; SY, symptomatic.

*vivax malaria.*Figure 6A shows that cell clusters identified by CyTOF as differentially abundant between *P.* *vivax* symptomatic individuals and healthy immune controls segregated in 2 main families. To explore how these populations associate with one another, Spearman’s correlation networks were applied. Clusters of activated, atypical, and classical CCR6/CXCR5-expressing IgM^+^IgD^+^ MBCs abundant among symptomatic individuals were positively correlated with high frequencies of TH1-like TFH cells (Figure 6B). A stronger association with CXCR3^+^ TFH cells was found with class-switched CCR6^+^ atypical MBCs, suggesting a relationship between TH1-polarized TFH cells and the induction of CCR6-expressing MBCs (Figure 6B). Similar results were found within cell clusters differentially abundant in individuals carrying *P*. *vivax* asymptomatic infections (Supplemental Figure 2). All populations of memory CD4^+^ T cells overrepresented in healthy immune controls were highly correlated with each other (Figure 6C). Among those cells, frequencies of TH1-like CD4^+^ T cells with features of TSCM cells, as described in Figure 4, were strongly correlated with CD27^lo^CD127^lo^CD25^+^ Treg cells. Subsets of TH2- and TH17-polarized cells together with 2 populations of TH2-like TFH cells (CD27^+^ and CD127^+^) were positively associated with class-switched activated, atypical, and classical MBCs. Together, these results suggest that whereas concomitant *P*. *vivax* infection results in the induction of TH1-like TFH cells that preferentially support the expansion of CCR6^+^ unswitched MBCs, protection from symptomatic infection is associated with class-switched MBCs that require help from populations of TH2-like TFH cells for their successful expansion.

The association of cell frequency with risk of experiencing *P*. *vivax* symptomatic and asymptomatic malaria was calculated. CXCR3^+^ TH1-like TFH cells, along with populations of CXCR5^+^CCR6^+^ unswitched classical and activated MBCs, were found among the most significant populations associated with increased risk of infection (Figure 6D). In contrast, various populations of class-switched MBCs expressing low levels of or no CXCR5 and CCR6, including T-bet^+^ atypical MBCs, were associated with reduced risk of infection. High frequencies of TH2-like TFH cells, TSCM-like TH1 cells, and TH2-polarized CD25^+^ Treg cells were among the strongest predictors of reduced risk of *P*. *vivax* malaria (Figure 6D).

Next, to identify cell populations specifically associated with protection from asymptomatic or symptomatic *P*. *vivax* infection, the automated machine learning workflow sequential iterative modeling overnight (SIMON) (47) was applied. Figure 6, E and F, show ROC curves assessing the performance of the 3 best performing models. This showed that each model was able to correctly classify individuals with either an asymptomatic or symptomatic infection with a high degree of accuracy (area under the ROC curve [AUROC] 0.71–0.96). Each feature assessed in the analysis was then assigned a variable importance score from 0 to 100 based on its contribution to the model. The ranking as well as the variable importance score for each feature were identical among the 3 best performing models. The top-ranked features (variable importance score > 85%) are shown in Figure 6, G and H. High frequencies of CD27^lo^CD25^+^CD127^lo^ TH2 Treg cells and class-switched activated MBCs, together with reduced frequencies of IgM^+^IgD^+^CXCR5^+^CCR6^+^T-bet^+^ activated MBCs and CXCR3^+^PD-1^+^T-bet^+^ TFH cells, best predicted protection from symptomatic infection (Figure 6G). TH2-like Treg cells were also a feature of protection from asymptomatic infection, along with high frequencies of CD127^+^ TH17 cells and TH1-like CD27^+^CD127^+^CD4^+^ T cells, with reduced frequencies of this lineage among the TH2 compartment (Figure 6H). Thus, these results suggest that whereas class-switched MBCs and humoral immunity are important for the control of symptomatic *P*. *vivax* malaria, cellular responses mediated by CD4^+^ T cells are required for the control of asymptomatic infection of low parasite burden.

## Discussion

IFN-γ–driven responses to malaria have long been viewed as a double-edged sword, because they have been shown to be involved in the control of parasitemia but also play a role in the development of clinical symptoms (48). Here, the use of high-dimensional single-cell mass cytometry allowed us to identify specific subsets of memory CD4^+^ T cells and MBCs expressing the TH1-defining transcription factor T-bet, associated with either increased or reduced risk of *P*. *vivax* infection, demonstrating that IFN-γ–driven responses to malaria support the development of diverse responses that have the potential to give rise to different infection outcomes. Our approach also identified specific populations of Treg cells, TH2-like TFH cells, TH17 cells, and MBCs required to support protective immune responses to infection.

Parasite-specific IgM^+^ MBCs have been detected in *P*. *falciparum*–exposed individuals (49), and IgM antibodies specific for blood-stage antigens have been found to be associated with protection from symptomatic malaria (50, 51). Unlike IgG^+^ MBCs, which differentiate into antibody-secreting cells during secondary responses, IgM^+^ MBCs appear to have the capacity to reenter GCs, where they undergo class-switch recombination and further rounds of somatic hypermutation to remodel their affinity for antigen (52–54). Aligned with this concept, mouse malaria studies found that IgM^+^ MBCs adopt a GC B cell phenotype upon secondary infection (55), while B cell receptor repertoire sequencing revealed that IgM^+^ MBCs acquire further mutations upon *P*. *falciparum* reinfection in children (56). Unlike those reports supporting a role for IgM^+^ MBCs in clinical immunity to *P*. *falciparum*, class-switched but not IgM^+^ MBCs were found to reduce the risk of *P*. *vivax* malaria. Protection from infection was not only associated with classical MBCs. Populations of class-switched atypical MBCs also predicted reduced risk of *P*. *vivax* malaria, suggesting that specific subsets within this cell lineage, previously viewed as a predictor of poor infection outcomes (38), might in fact play a beneficial role in the control of parasite burden. The strong correlation between frequencies of class-switched MBCs and subsets of TH2-polarized TFH cells suggests that these cells provide help for the development of these protective responses to infection.

Expression of the TH1 transcription factor T-bet has been detected in atypical MBCs from malaria-exposed individuals (34, 35, 38). Increased frequencies of T-bet^hi^ atypical MBCs were found to be associated with frequent febrile episodes (38). Here, using unsupervised high-dimensional data analysis, we identified different populations of T-bet^+^ atypical MBCs predicting increased or reduced risk of *P*. *vivax* infection depending on the expression of the chemokine receptor CCR6. CCR6^+^ cells associated with poor infection outcomes also expressed high levels of the Fc-like inhibitory receptor FcRL5, which has been found to be upregulated among MBCs with reduced capacity to differentiate into antibody-secreting cells (34, 35). CCR6 has recently been identified as a reliable marker of MBC precursors that emerge with low affinity for antigen from both mouse and human GCs (57). Thus, it is conceivable that atypical MBCs expressing high levels of CCR6 found in individuals carrying *P*. *vivax* infection might constitute a population of low-affinity MBCs with poor effector capacity rapidly emerging from GC reactions in response to infection. Furthermore, our findings identified expression levels of CCR6 rather than T-bet, not only on atypical but also on classical MBCs, as a good predictor of increased risk of *P*. *vivax* malaria.

The induction of TH1-like CXCR3^+^ TFH cells was strongly correlated with increasing frequencies of IgM^+^IgD^+^ MBCs. Consistent with previous studies (31), circulating CXCR3^+^ TFH cells were overrepresented among individuals with symptomatic *P*. *vivax* infection. Interestingly, these cells were also found in individuals with asymptomatic infection, suggesting that low parasite burdens are sufficient to induce the activation of this pathway. In contrast, a population of TH1 memory CD4^+^ T cells was identified as a strong predictor of reduced risk of infection. Although no functional assays were done in this study to confirm this proposition, these cells expressed high levels of several markers, including CXCR3, CD27, CD127, and CCR7, resembling features of a TSCM phenotype. TSCMs possess self-renewal ability and the multipotent capacity to reconstitute different lineages of T cell subsets. These cells, which persist at stable levels in circulation after exposure to antigen (58), have been recently identified in response to viral, bacterial, and parasitic infections (59–61), with associations with favorable infection outcomes. Whether these findings reflect the capacity of TSCMs to rapidly differentiate into effector T cells to control infection is still unclear but raise the possibility that in response to malaria, TSCMs might contribute to protection from infection via multiple mechanisms, including providing help for antibody formation or secreting IFN-γ required for clearance of parasitized red blood cells. In support of this proposition, studies in rodent malaria identified CXCR5^–^ TH1-like memory CD4^+^ T cells that have B cell helper capacity during recall responses (32). Furthermore, CD4^+^ T cells were also found to be an important source of IFN-γ in *P*. *vivax* infection (62) and contribute to the destruction of infected erythrocytes.

High levels of IFN-γ have also been observed in patients with severe *P*. *vivax* malaria (63). Thus, favorable infection outcomes may require IFN-γ responses to be regulated to maintain the balance between protective immune responses and immunopathology in malaria. In this cohort, increased frequencies of Tregs were observed in immune healthy controls compared with asymptomatic and symptomatic *P*. *vivax*–infected individuals. Studies in *P*. *falciparum* have shown that CD25^+^CD127^+^FoxP3^+^ Tregs that are induced in response to infection can suppress TH1 cell function in an antigen-specific manner (64). Aligned with those findings, we observed a strong correlation between frequencies of Treg cells and CD27^+^CD127^+^CCR7^+^ TH1 memory CD4^+^ T cells, suggesting the Treg cell pool may expand to regulate IFN-γ production elicited by a TH1 memory compartment to promote parasite clearance while preventing the development of severe disease in immune healthy controls.

Previous studies found an expansion of CD4^+^ T cells producing IL-17 triggered by uncomplicated *P*. *vivax* malaria, which correlates with the number of CD4^+^ T cells producing IFN-γ, IL-10, and TGF-β in response to infection (65). Our unsupervised mass cytometry approach followed by machine learning identified a population of CD127^+^ TH17 CD4^+^ T cells, highly correlated with TH1 memory CD4^+^ T cells, as a strong predictor of protection from asymptomatic infection. Thus, together these results encourage new mechanistic studies to uncover processes by which the TH17 pathway contributes to the control of uncomplicated malaria.

Although cellular immune responses induced by symptomatic and asymptomatic *P*. *vivax* malaria were similar, machine learning approaches were able to dissect different populations predicting reduced risk of high or low parasitemia infection. Whereas class-switched but not IgM^+^IgD^+^ MBCs appeared as important predictors of reduced risk of symptomatic infection, the presence of Treg cells along with TH1 and TH17 CD4^+^ T cell responses were features of protection from asymptomatic *P*. *vivax* malaria. Thus, our results support the notion that whereas B cell–mediated antibody responses participate in the induction of clinical immunity, activation of cellular immune responses might be necessary to provide protection from asymptomatic infection of low parasite burden. Previous studies have found associations between antibodies against the vaccine candidate DBP and protection from symptomatic *P*. *vivax* infection in Papua New Guinean children (14, 66). Although the present study did not detect associations between DBP-specific IgG and risk of symptomatic infection, a trend toward increased number of MBCs specific for this antigen was observed in healthy immune controls and asymptomatic individuals (data not shown). Antibodies against a range of *P. vivax* blood-stage antigens, including merozoite surface protein-3 (MSP-3), MSP-9, erythrocyte binding protein, and reticulocyte binding protein 2b, have been shown to be associated with protection in various geographical regions (14, 67–70). Further work will be required to determine the overall breadth of antibody responses required for protection against *P*. *vivax* symptomatic infection.

## Methods

### Study design.

A cross-sectional study was conducted in the Timika region of Papua, Indonesia, in July to December 2014. Malaria transmission in Timika is high, with an estimated incidence of malaria of 512 and 322 per 1000 population per year for *P*. *falciparum* and *P*. *vivax*, respectively (71). Consenting participants (aged between 5–46 years) donated a 10 mL venous blood sample at enrollment, and PBMCs and plasma were frozen. Parasite densities and the species of infection were determined by light microscopy examination of Giemsa-stained blood smears. In addition, 40 symptomatic patients presenting with malaria at the Rumah Sakit Mitra Masyarakat Hospital were enrolled in the study between November and December 2014. Participants with light microscopy–confirmed malaria infections were administered first-line antimalarial treatment according to the Indonesian Ministry of Health guidelines after sample collection. Hemoglobin, hematocrit, WBC count, and platelet count were measured for each participant using a hematology analyzer. Genomic DNA was extracted from dried blood spots using the QIAamp DNA Blood Mini Kit (QIAGEN). The presence of *Plasmodium* species was confirmed by a species-specific nested PCR assay as previously described (72). Symptomatic malaria cases were defined as individuals with an axillary fever at least 37.5°C, chills, malaise, headache, or vomiting at the time of examination or up to 24 hours prior to the examination and the presence of a *P*. *vivax*–positive blood smear and no other cause of fever discernible by physical exam. All individuals included in the immunity study were Papuan. Symptomatic individuals included in the immunity study had more than 150 parasites/μL blood whereas individuals with a *P*. *vivax*–positive blood smear and no clinical symptoms were classified as having asymptomatic infections. Healthy immune controls had a negative light microscopy and PCR diagnosis. Previous exposure to malaria in these individuals was confirmed by ELISA against DBP recombinant protein.

### ELISA.

ELISA plates (Corning) were coated by overnight incubation with 0.5 μg/mL of Duffy binding protein region III-V (DBP RIII-V) in carbonate buffer pH 9.6 at 4°C overnight. Recombinant DBP RIII-V was expressed and purified as described elsewhere (15, 73). After washing with phosphate-buffered saline (PBS; Gibco), plates were blocked with 5% skim milk in PBS for 1 hour at 37°C before incubation with serial 2-fold dilutions of plasma samples for 1 hour at 37°C. Plates were then washed 3 times with 0.05% Tween-20 in PBS (PBST) and incubated with HRP-conjugated anti-human IgG antibodies (clone JDC-10; Southern Biotech) for 1 hour at 37°C. After washing with PBST, bound complexes were detected by reaction with tetramethylbenzidine substrate (KPL). The reaction was stopped with 1 M phosphoric acid, and absorbance was measured at 450 nm. Plasma samples from malaria-naive, anonymous, Australian blood donors were included as negative controls. Antibody titers were calculated as the reciprocal of the plasma dilution with an optical density value higher than the mean of malaria-naive control individuals plus 2 standard deviations.

### CyTOF.

PBMCs (1× 10^6^ to 2 × 10^6^) from *P*. *vivax* symptomatic (*n* = 11) and asymptomatic (*n* = 19) infected individuals as well as healthy immune controls (*n* = 24) were stained with 5 μM Cell-ID Cisplatin (Fluidigm) in PBS for 5 minutes at room temperature. Cells were then blocked with Human TruStain FcX (BioLegend) and stained with a cocktail of surface marker antibodies (Supplemental Table 1) in CyTOF staining buffer (PBS with 0.5% bovine serum albumin from MilliporeSigma and 0.02% sodium azide from MilliporeSigma) for 30 minutes at room temperature. Metal-labeled anti-fluorochrome antibodies were used to detect staining with APC- and PE-conjugated antibodies. After surface staining, cells were fixed and permeabilized with a Maxpar nuclear antigen staining buffer set (Fluidigm) and then stained with a 161Dy-conjugated anti–T-bet (clone 4B10; Fluidigm) antibody for 45 minutes at room temperature. Cells were then washed twice and stored in Maxpar fix and perm buffer (Fluidigm) with 125 nM Cell-ID iridium intercalator (Fluidigm) for a minimum of 18 hours. Prior to data acquisition, cells were washed twice by centrifugation at 800*g* for 5 minutes in ultrapure water and then resuspended in a 1:10 dilution of 4-Element EQ normalization beads (Fluidigm) in ultrapure water. Cells were analyzed on a Helios model mass cytometer (Fluidigm) at approximately 300 events/s. Data were normalized using the signal from 4-Element EQ Beads (Fluidigm) as previously described (74). Manual gating was then performed using FlowJo version 10 (BD Biosciences) to exclude doublets and dead cells, before individual cell populations were selected and exported for further analysis in Cytobank (75). Individual cell populations were then visualized using viSNE (76), while FlowSOM (77) was used to identify cell subpopulations. The following parameters were included in the viSNE analysis for classical, atypical, and activated MBCs: IgD, IgM, CXCR5, CXCR3, CCR6, CD45RA, PD-1, and T-bet. For TH1-, TH2-, and TH17-like CD4^+^ T cells, the following parameters were included: CXCR5, PD-1, CD27, CD25, CD127, and T-bet. For TFH cells, the following parameters were included: CXCR3, CCR6, PD-1, CD27, CD25, CD127, and T-bet. Self-organizing maps were then generated for each cell population using hierarchical consensus clustering on the t-SNE axes.

### Flow cytometry.

PBMCs (1 × 10^6^ to 2 × 10^6^) were stained with a 1:1000 dilution of Fixable Viability Dye eFluor 506 (eBioscience) in PBS for 25 minutes on ice. Cells were then with blocked with Human TruStain FcX (BioLegend) and stained with a cocktail of surface marker antibodies (Supplemental Table 2) in staining buffer (PBS with 1% HI-FBS and 2 mM ethylenediaminetetraacetic acid) for 25 minutes on ice. After surface staining, cells were fixed and permeabilized using the FoxP3/transcription factor staining buffer set (eBioscience) and then stained with BV421- or PE-Cy7–conjugated T-bet (clone 4B10; BioLegend) and BB700-conjugated FoxP3 (clone 236/E7; BD) antibodies for 50 minutes at room temperature. Cells were then washed twice, resuspended in staining buffer, and analyzed on a BD Biosciences Fortessa X-20. Data analysis was performed using FlowJo version 10.

### SIMON.

To define minimum sets of features capable of classifying each of the *P*. *vivax* clinical presentations in an unsupervised manner, SIMON, an automated machine learning approach for clinical data feature selection (47), was applied to the cell populations identified by CyTOF. Briefly, data were partitioned into training and test sets as outlined in Table 1. Samples in each set were balanced to ensure even category distribution. Five of the most commonly used machine learning algorithms were selected for model training: svmLinear2, naive_bayes, pcaNNet, LogitBoost, and hdda. Predictor accuracy was first assessed on the training sets by 10-fold cross-validation performed 5 times and subsequently assessed on the test set to prevent overfitting. Model performance was judged by accuracy, specificity, sensitivity, and AUROC, calculated for both train and test sets. Features were assigned a variable importance score (from 0 to 100) based on their contribution to these performance metrics.

### Statistics.

Univariate tests between groups were conducted using Prism version 8 (GraphPad Software Inc). An unpaired, 2-tailed *t* test or 1-way ANOVA with a Holm-Sidak multiple-comparison test was used if normality was not rejected (D’Agostino-Pearson normality test); otherwise, the Mann-Whitney *U* test or Kruskal-Wallis with Dunn’s multiple-comparison test was used. Multivariate analyses were conducted using R. Correlations between relative frequencies of cell populations were determined using Spearman’s rank correlation, and correlation networks were visualized using the corrr package. Linear regression models were fitted using the limma package in R (78), and differential abundance was assessed using moderated 2-tailed *t* tests, a robust trended empirical Bayes procedure (79). The FDR was controlled below 0.05 using the method of Benjamini and Hochberg (80). Log_2_ fold changes and confidence intervals were exported from limma. Logistic regression models were fitted for pairwise comparisons between groups to determine the odds ratio for each cell population. A *P* value less than 0.05 was considered significant.

### Study approval.

This study was approved by the human research ethics committees of the Eijkman Institute for Molecular Biology, the Walter and Eliza Hall Institute of Medical Research, the Northern Territory Department of Health & Families (Darwin, Northern Territory, Australia), and the Menzies School of Health Research. Written informed consent was obtained from all study participants prior to their inclusion in the study.

## Author contributions

LJI and DSH conceived and designed the study. AMP, DA, FNC, JRP, EK, PP, NS, LT, RNP, BA, and RN conducted the field sample collection. LJI, HMP, AL, RASU, and EME performed the experiments. JH and AFC provided reagents. LJI, RASU, SIS, WA, CSNLWS, DS, GKS, and DSH performed the analysis. LJI and DSH wrote the manuscript. DSH supervised the study.

## Supplementary Material

Supplemental data

## Figures and Tables

**Table 1:**
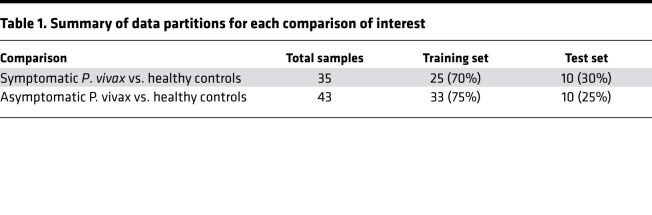
Summary of data partitions for each comparison of interest

## Version 1 06/15/2021

In-Press Preview

## Version 2 07/22/2021

Electronic publication
