# Supplementary material for: High-dimensional mass cytometry identifies T cell and B cell signatures predicting reduced risk of Plasmodium vivax malaria
Source: JCI Insight. 2021 Jul 22;6(14):e148086. doi: 10.1172/jci.insight.148086 (PMC8410020; doi:10.1172/jci.insight.148086)
Supplement: Supplemental data [file jciinsight-6-148086-s130.pdf]

Supplemental Material

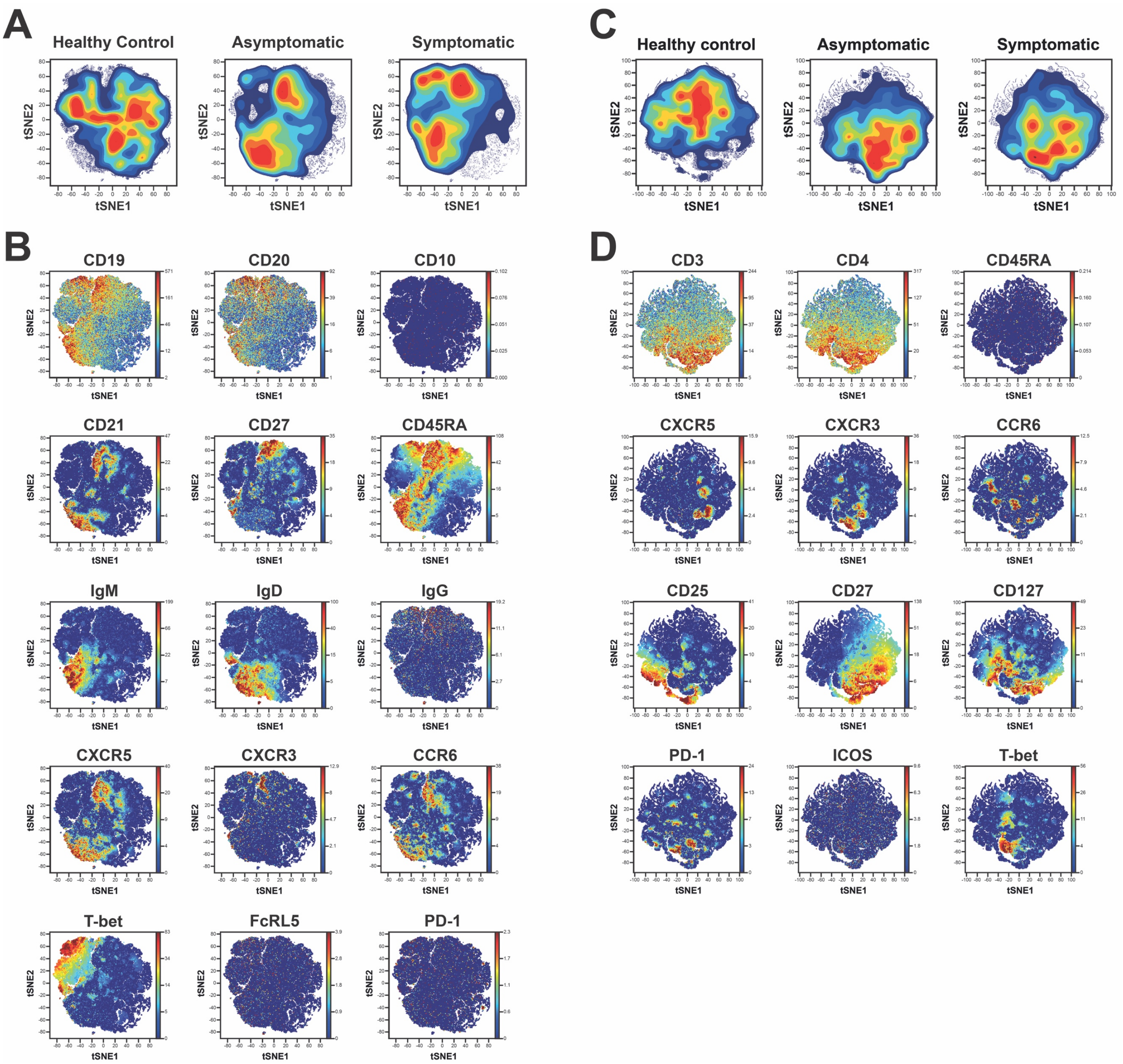

Supplemental Figure 1: Visualization of marker expression among the entire MBC and memory CD4$^+$ T cell compartments. PBMCs from *P. vivax* symptomatic (n=11) and asymptomatic (n=19) infected individuals as well as healthy immune controls (n=24) were stained with a panel of metal-labelled antibodies and analysed by CyTOF. tSNE analysis was performed on the entire MBC (**A**) and memory CD4$^+$ T cell compartments (**C**). tSNE plots display cell density and represent the pooled data for each group. Each marker used for the characterization of MBCs and memory CD4$^+$ T cells was then overlaid on tSNE plots (**B** and **D**).

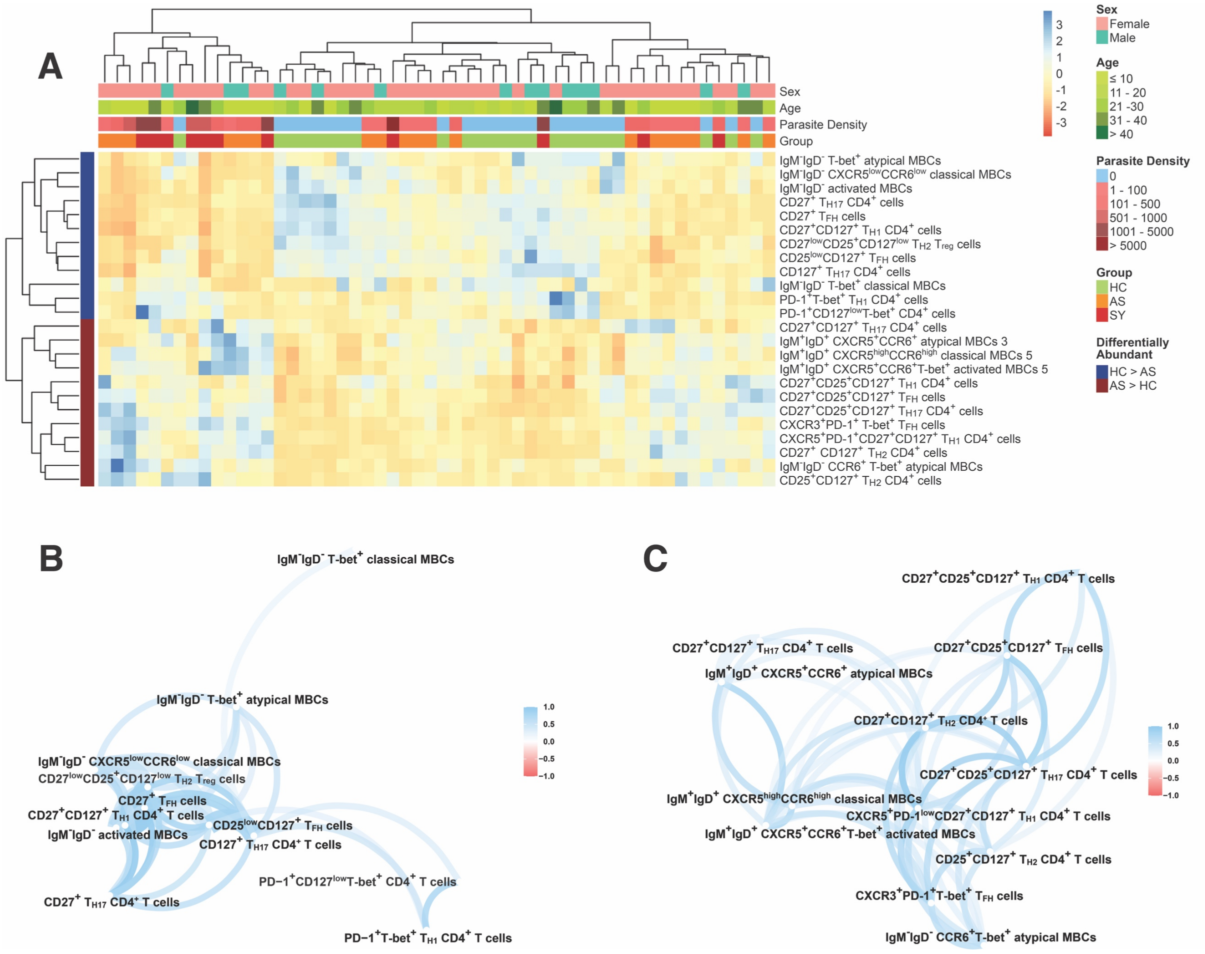

Supplemental Figure 2: Correlations between cell populations that are differentially abundant between healthy controls and asymptomatic individuals. PBMCs from *P. vivax* symptomatic (n=11) and asymptomatic (n=19) infected individuals as well as healthy immune controls (n=24) were stained with a panel of metal-labelled antibodies and analysed by CyTOF. Unsupervised hierarchical clustering heatmap showing the frequency of differentially abundant cell populations between healthy controls and asymptomatic individuals (**A**). Spearman correlation networks were used to determine the relationship between cell populations that were either reduced (**B**) or increased (**C**) in healthy controls compared to individuals with an asymptomatic infection.

Supplemental Table 1: Surface marker antibodies for CyTOF

Antibody	Clone	Supplier
141Pr-conjugated anti-CCR6	11A9	Fluidigm
143Nd-conjugated anti-CD45RA	HI100	Fluidigm
146Nd-conjugated anti-IgD	IA6-2	Fluidigm
147Sm-conjugted anti-CD20	2H7	Fluidigm
151Eu-conjugated anti-ICOS	DX29	Fluidigm
152Sm-conjugated anti-CD21	BL13	Fluidigm
153Eu-conjugated anti-CXCR5	RF8B2	Fluidigm
156Gd-conjugated anti-CXCR3	G025H7	Fluidigm
158Gd-conjugated anti-CD10	HI10a	Fluidigm
165Ho-conjugated anti-CD19	HIB19	Fluidigm
167Er-conjugated anti-CD27	L128	Fluidigm
170Er-conjugated anti-CD3	UCHT1	Fluidigm
172Yb-conjugated anti-IgM	MHM-88	Fluidigm
174Yb-conjugated anti-CD4	SK3	Fluidigm
175Lu-conjugated anti-PD-1	EH12.2H7	Fluidigm
176Yb-conjugated anti-CD127	A019D5	Fluidigm
APC-conjugated anti-IgG	M1310G05	Biolegend
PE-conjugated anti-FcRL5	509f6	Biolegend
145Nd-conjugated anti-PE	PE001	Fluidigm
162Dy-conjugated anti-APC	APC003	Fluidigm

Supplemental Table 2: Surface marker antibodies for FACS

Antibody	Clone	Supplier
BB515-conjugated anti-CCR6	11A9	BD
PE-conjugated anti-CCR4	1G1	BD
PE CF594-conjugted anti-CXCR3	1C6/CXCR3	BD
Pacific Blue-conjugated anti-CD25	M-A251	Biolegend
AF647-conjugated anti-CXCR5	RF8B2	BD
AF532-conjugated anti-CD3e	UCHT1	Invitrogen
APC-Cy7-conjugated anti-CCR7	G043H7	Biolegend
BV605-conjugated anti-CD127	HIL-7R-M21	BD
BV650-conjugated anti-CD27	MT2T4	BD
BV711-conjugated anti-PD-1	EH12.2H7	Biolegend
BV786-conjugated anti-CD4	SK3	BD
PE-Cy5-conjugated anti-CD45RA	HI100	BD
FITC-conjugated anti-IgA	Polyclonal	SouthernBiotech
Pacific Blue-conjugated anti-CCR6	G034E3	Biolegend
APC-Fire 750-conjugated anti-IgD	IA6-2	Biolegend
PE-conjugated anti-FcRL5	509f6	Biolegend
PE-Cy7-conjugated anti-CD21	Bu32	Biolegend
APC-conjugated anti-IgG	M1310G05	Biolegend
AF700-conjugated anti-CD10	HI10a	BD
PerCP-Cy5.5-conjugated anti-IgE	MHE-18	Biolegend
BV605-conjugated anti-IgM	MHM-88	Biolegend
BV711-conjugated anti-CD19	HIB19	Biolegend
BV785-conjugated anti-CD20	2H7	Biolegend
